# Supplementary material for: Development of the Aerial Remote Triage System using drones in mass casualty scenarios: A survey of international experts
Source: PLoS One. 2021 May 11;16(5):e0242947. doi: 10.1371/journal.pone.0242947 (PMC8112676; doi:10.1371/journal.pone.0242947)
Supplement: S1 Appendix — (DOCX) [file pone.0242947.s001.docx]

**S1 Appendix. Questionnaire for experts: algorithm assessment**

**English version**

Dear colleague:

Professionals from Emergency Medical Drone and the research group Nursing and Innovation in Health Care (CuiDsalud) from the University of Jaén are developing the Project Drones for Triage in Medical Emergencies. Our first objective is to develop and validate a procedure for remote triage using Unmanned Aircrafts (drones). In order to achieve this, we ask you to fill out a questionnaire. We consider that your knowledge can be of great help for the development of this project.

The questionnaire consists in two blocks of statements. The first one is composed of general statements about the use of drones in health emergencies. The second contains statements related to the Aerial Remote Triage System (ARTS). We ask you to show the agreement with the statements according to the following scale:

1. Completely disagree
2. Partially disagreeing
3. Partially in agreement
4. Completely in agreement

It is very important that you give us reasons for your partial or total disagreement with the statements, providing suggestions for improvement. There are spaces designed after each statement to do it.

You can find more information about the project at: <https://cuidsalud.com/en/inv/drones-emergencies/>

We request that you do not disseminate information regarding the Aerial Remote Triage System until its final version is published. Once it is published, we will send you the relevant information so that you can use or disseminate it, if you wish.

If you have any questions or suggestions, please contact us at the following e-mail address: cagarcia@ujaen.es

If you respond to the questionnaire, you give you consent for the data reported to be used in a study.

The estimated time it will take you to complete the questionnaire is 10 minutes. Thank you very much for your collaboration!

*
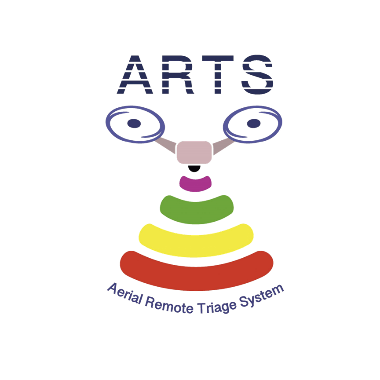
*

**SOCIO-DEMOGRAPHIC DATA**

Before you start to assess the statements, we ask you for some information regarding your training and experience. This information will be used only for the purpose of characterizing the sample of experts who are participating in the evaluation and improvement of the Aerial Remote Triage System. The data are anonymous and their confidentiality is guaranteed.

Degree(s): □ Medicine □ Nursing □ Other (specify)

Work field: □ Assistance □ Management □ Academic □ Other (specify)

Years of professional experience in emergencies and/or disasters:

**SECTION 1**

**Use of Drones in Health Emergencies**

Please indicate your agreement with the following general statements related to the use of drones in health emergencies according to the scale shown below them. It is very important that you give us reasons for your partial or total disagreement with the statements, providing suggestions for improvement.

**1. The deployment of unmanned aircraft in complex health emergencies scenarios (nuclear, radiological, biological, chemical, terrorist attacks, active shooting, suicide bombs) or without initial ground access, can help in the first evaluation of the scenario (type of incident, added risks, number of patients and their distribution in the place, access and evacuation routes).**

□ Completely in agreement □ Partially in agreement □ Partially disagreeing □ Completely disagree

| Comments |
| --- |

**2. The use of drones in complex health emergencies (nuclear, radiological, biological, chemical, terrorist attacks, active shooting, suicide bombs) or in hard-to-reach areas may be a valid option and preferable to physical access at the initial stage of the emergency.**

□ Completely in agreement □ Partially in agreement □ Partially disagreeing □ Completely disagree

| Comments |
| --- |

**3. The collection of relevant information for clinical assessment through cameras, sensors and on-board systems in drones flying over complex health emergency scenarios may be considered as an alternative when ground access is not feasible.**

□ Completely in agreement □ Partially in agreement □ Partially disagreeing □ Completely disagree

| Comments |
| --- |

**4. The information provided by cameras, sensors and on-board systems in drones flying over complex health emergency scenarios can be very useful in the decision-making process for the management of the emergency and the assistance to the patients involved.**

□ Completely in agreement □ Partially in agreement □ Partially disagreeing □ Completely disagree

| Comments |
| --- |

**5. A drone with speakers can provide remote medical support to patients by means of indications broadcast through the speakers.**

□ Completely in agreement □ Partially in agreement □ Partially disagreeing □ Completely disagree

| Comments |
| --- |

**6. A drone with speakers can communicate support messages to the patients in complex health emergencies where access is impossible.**

□ Completely in agreement □ Partially in agreement □ Partially disagreeing □ Completely disagree

| Comments |
| --- |

**7. A drone with speakers can ask all persons involved in the emergency who are able to walk (by-standers) to remain standing, along with others who are not able to do so, in order to try to help them, if they consider themselves capable of helping.**

□ Completely in agreement □ Partially in agreement □ Partially disagreeing □ Completely disagree

| Comments |
| --- |

**8. A drone with speakers allows instructions for self-protections to be given to people who are unable to walk.**

□ Completely in agreement □ Partially in agreement □ Partially disagreeing □ Completely disagree

| Comments |
| --- |

**9. A drone with speakers can be used to instruct a by-stander to place a patient in the recovery position or to employ (or self-employ) some form of haemostatic technique to potentially exsanguinating wounds.**

□ Completely in agreement □ Partially in agreement □ Partially disagreeing □ Completely disagree

| Comments |
| --- |

**10. A drone can carry useful medical equipment to help in an emergency situation such as medication (antidote auto-injectors, analgesia), bleeding kit, isothermal sheet...before the arrival of the first responders.**

□ Completely in agreement □ Partially in agreement □ Partially disagreeing □ Completely disagree

| Comments |
| --- |

**11. A drone deployed flying over a complex health emergency scenario allows individual assessment of patients and care prioritization through images and speakers.**

□ Completely in agreement □ Partially in agreement □ Partially disagreeing □ Completely disagree

| Comments |
| --- |

**SECTION 2**

**Assessment of the Aerial Remote Triage System (ARTS)**

Please show your degree of agreement with the following statements related to the Aerial Remote Triage System according to the scale shown below them. It is very important that you give us reasons for your partial or total disagreement with the statements, providing suggestions for improvement.

The complete algorithm is shown below to guide you in your answers. The assessment will end when the patient is assigned the corresponding colour according to this algorithm.


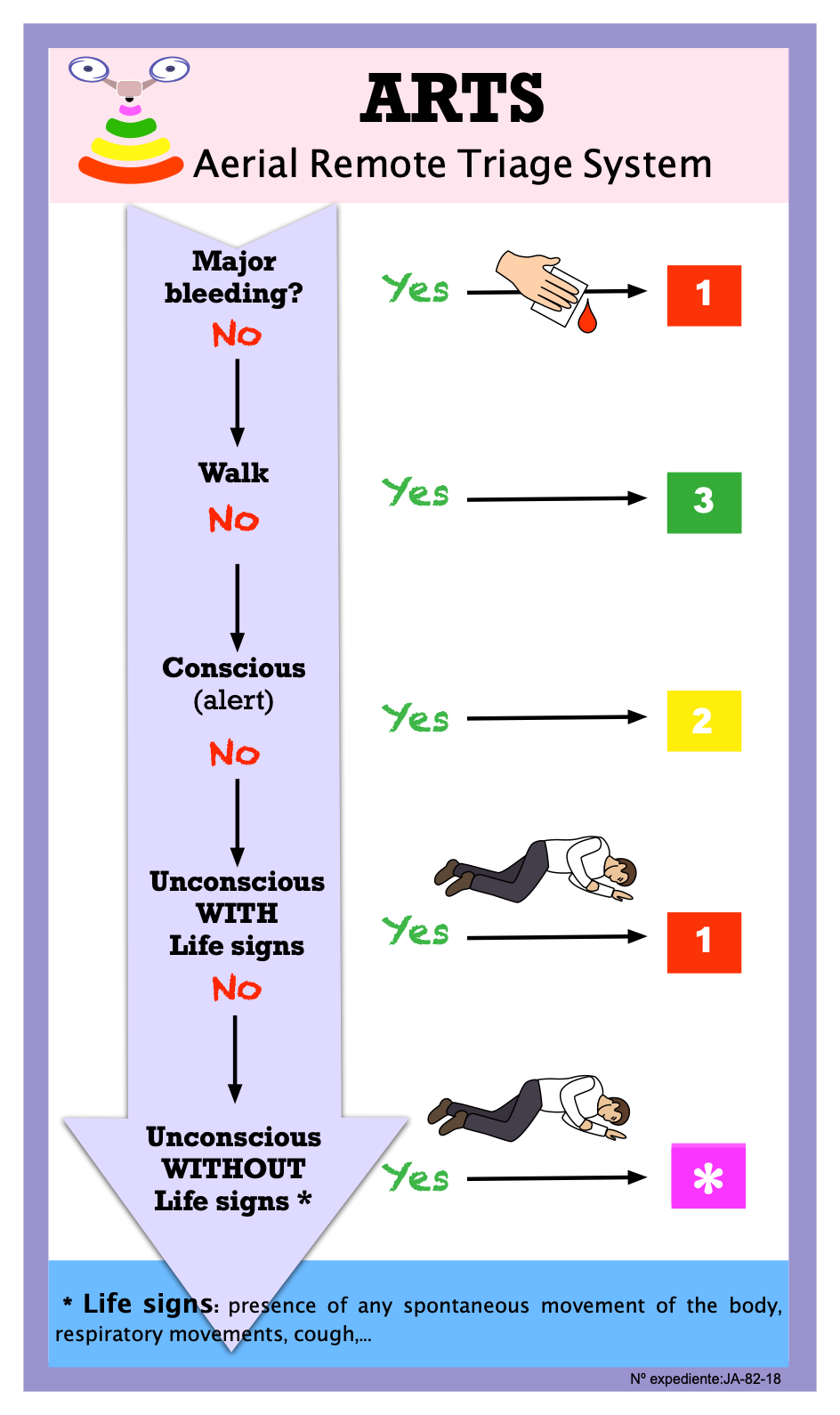


**1. The first step before starting the triage will be to broadcast an audio message through the drone speakers instructing people who can walk to remain standing and those who cannot walk to make some movement.**

□ Completely in agreement □ Partially in agreement □ Partially disagreeing □ Completely disagree

| Comments |
| --- |

**2. The assessment of care priority will start with those patients who do not respond to messages from the drone speakers.**

□ Completely in agreement □ Partially in agreement □ Partially disagreeing □ Completely disagree

| Comments |
| --- |

**3. In the assessment of care priority supported by drones, the aspects to be evaluated would be, in this order: 1- Significant bleeding; 2- Walking, 3- Aware (awake), 4- Sign of life.**

□ Completely in agreement □ Partially in agreement □ Partially disagreeing □ Completely disagree

| Comments |
| --- |

**4. If in the assessment of care priority supported by drones the patient present wounds with important bleeding (exsanguinating), priority 1 (RED) will be assigned.**

□ Completely in agreement □ Partially in agreement □ Partially disagreeing □ Completely disagree

| Comments |
| --- |

**5. If in the assessment of care priority supported by drones the patient present wounds with important bleeding (exsanguinating according to criteria of Hartford Consensus), the employment of some form of haemostatic technique will be instructed to the patient or some by-stander through the drone speakers, if possible.**

□ Completely in agreement □ Partially in agreement □ Partially disagreeing □ Completely disagree

| Comments |
| --- |

**6. If in the assessment of care priority supported by drones the patient can walk, priority 3 (GREEN) will be assigned.**

□ Completely in agreement □ Partially in agreement □ Partially disagreeing □ Completely disagree

| Comments |
| --- |

**7. If in the assessment of care priority supported by drones the patient does not walk, but is aware (awake: responds to audio stimuli), priority 2 (YELLOW) will be assigned.**

□ Completely in agreement □ Partially in agreement □ Partially disagreeing □ Completely disagree

| Comments |
| --- |

**8. If in the assessment of care priority supported by drones the patient is apparently unaware but shows signs of life (spontaneous movements of the body, respiratory movements, coughing...), priority 1 (RED) will be assigned.**

□ Completely in agreement □ Partially in agreement □ Partially disagreeing □ Completely disagree

| Comments |
| --- |

**9. If in the assessment of care priority supported by drones the patient is apparently unconscious and with signs of life, by-standers will be instructed through drone speakers to place the patient in the recovery position, if possible.**

□ Completely in agreement □ Partially in agreement □ Partially disagreeing □ Completely disagree

| Comments |
| --- |

**10. If in the assessment of care priority supported by drones the patient is apparently unconscious and without signs of life (spontaneous movements of the body, respiratory movements, coughing...), priority * (VIOLET) will be assigned.**

□ Completely in agreement □ Partially in agreement □ Partially disagreeing □ Completely disagree

| Comments |
| --- |

**11. In the assessment of care priority supported by drones, the PRIORITY * (VIOLET) indicate that the patient is waiting for re-assessment by the first responders.**

□ Completely in agreement □ Partially in agreement □ Partially disagreeing □ Completely disagree

| Comments |
| --- |

**12. If in the assessment of care priority supported by drones no signs of life can be observed in the patient (spontaneous movements of the body, respiratory movements, coughing...), by-standers will be instructed through drone speakers to place the patient in the recovery position, if possible.**

□ Completely in agreement □ Partially in agreement □ Partially disagreeing □ Completely disagree

| Comments |
| --- |

**13. Providing the first responders with the results of the Aerial Remote Triage System before they have access to the site, can be very useful and helpful.**

□ Completely in agreement □ Partially in agreement □ Partially disagreeing □ Completely disagree

| Comments |
| --- |

**Spanish version**

Estimado/a compañero/a:

Profesionales de Emergency Medical Drone y el grupo de investigación Enfermería e innovación en cuidados de salud (CuiDsalud) de la Universidad de Jaén estamos desarrollando el proyecto **Drones para el Triaje en Emergencias Sanitarias**. Nuestro primer objetivo es elaborar y validar un procedimiento de triaje remoto mediante vehículos aéreos no tripulados (drones). Para conseguirlo, hemos pensado en solicitar su participación como experto/a a través de la cumplimentación de este cuestionario. Consideramos que su conocimiento puede ser de gran ayuda para el desarrollo de este proyecto.

A continuación se muestran dos bloques de enunciados. En el primero aparecen una serie de enunciados generales acerca del uso de drones en emergencias sanitarias. En el segundo se plantean cuestiones relativas al prototipo de algoritmo del Sistema de Triaje Aéreo a Distancia. Le solicitamos que muestre su acuerdo con los enunciados según la siguiente escala:

1. Completamente en desacuerdo
2. Parcialmente en desacuerdo
3. Parcialmente de acuerdo
4. Completamente de acuerdo

Es muy importante que nos razone su desacuerdo parcial o total con los enunciados, aportando sugerencias de mejora, en los campos de comentarios tras cada enunciado.

Puede encontrar más información sobre el proyecto en: <https://cuidsalud.com/inv/drones-triage/>

Le solicitamos que **no difunda información relativa al algoritmo del Sistema de Triaje Aéreo a Distancia** hasta que no esté publicada su versión final. Una vez publicada la misma, le remitiremos la información pertinente para que pueda usarla o difundirla, si lo desea.

Para cualquier duda o sugerencia puede dirigirse a la siguiente dirección de correo electrónico: cagarcia@ujaen.es

Tiempo estimado 10 minutos. ¡Muchas gracias por su colaboración!


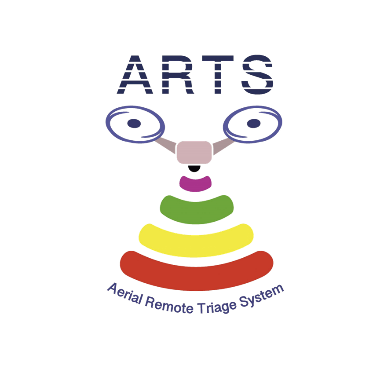


**DATOS SOCIODEMOGRÁFICOS**

Antes de comenzar a valorar los enunciados, le solicitamos alguna información relativa a su formación y experiencia. Esta información se usará tan solo con el objetivo de caracterizar a la muestra de expertos que participan en la valoración y mejora del algoritmo del Sistema de Triaje Aéreo a Distancia. Los datos compartidos serán en todo momento anónimos y se tratarán de manera desagregada.

Titulación: □ Medicina □ Enfermería □ Otra (especifique)

Ámbito de trabajo: □ Asistencial □ Gestión □ Académico □ Otro (especifique)

Años de experiencia profesional en el área de Emergencias sanitarias y/o Urgencias:

**SECCIÓN 1**

**Uso de Drones en Emergencias Sanitarias**

Por favor, muestre su grado de acuerdo con los siguientes enunciados generales sobre el uso de drones en emergencias sanitarias según la escala mostrada tras los mismos. Es muy importante que nos razone su desacuerdo parcial o total con los enunciados, aportando sugerencias de mejora, en los campos de comentarios tras cada enunciado.

1. **El despliegue de aeronaves no tripuladas (drones) en escenarios complejos de emergencias sanitarias (nucleares, radiológicos, biológicos, químicos, atentados terroristas, tiroteos activos, bombas suicidas) o sin acceso terrestre inicial, puede ayudar en la primera evaluación del escenario (tipo de incidente, riesgos añadidos, número de pacientes y su distribución en el lugar, rutas de acceso y evacuación).**

◻ 4 Completamente de acuerdo ◻ 3 Parcialmente de acuerdo ◻ 2 Parcialmente en desacuerdo ◻ 1 Completamente en desacuerdo

| Comentarios |
| --- |

1. **El uso de drones en emergencias sanitarias complejas (nucleares, radiológicas, biológicas, químicas, atentados terroristas, tiroteos activos, bombas suicidas) o en zonas de difícil acceso, puede ser una opción válida, en los momentos iniciales, y preferible al acceso físico.**

◻ 4 Completamente de acuerdo ◻ 3 Parcialmente de acuerdo ◻ 2 Parcialmente en desacuerdo ◻ 1 Completamente en desacuerdo

| Comentarios |
| --- |

1. **La recopilación de información relevante para la valoración clínica a través de las cámaras, sensores y sistemas embarcados en drones desplegados en escenarios complejos de emergencias sanitarias podrá plantearse como alternativa cuando no sea viable el acceso terrestre para tales fines.**

◻ 4 Completamente de acuerdo ◻ 3 Parcialmente de acuerdo ◻ 2 Parcialmente en desacuerdo ◻ 1 Completamente en desacuerdo

| Comentarios |
| --- |

1. **La información aportada a través de cámaras, sensores y sistemas embarcados en drones desplegados en escenarios complejos de emergencias sanitarias, puede resultar de gran utilidad en el proceso de toma de decisiones para la gestión de la emergencia y la asistencia a los pacientes involucrados.**

◻ 4 Completamente de acuerdo ◻ 3 Parcialmente de acuerdo ◻ 2 Parcialmente en desacuerdo ◻ 1 Completamente en desacuerdo

| Comentarios |
| --- |

1. **Un dron con sistema de megafonía puede ofrecer soporte médico remoto a las pacientes o personas heridas mediante indicaciones emitidas a través de este sistema.**

◻ 4 Completamente de acuerdo ◻ 3 Parcialmente de acuerdo ◻ 2 Parcialmente en desacuerdo ◻ 1 Completamente en desacuerdo

| Comentarios |
| --- |

1. **Un dron con sistema de megafonía permite comunicar expresiones de apoyo y solidaridad a los pacientes y heridos en emergencias complejas en lugares en los que es imposible el acceso de personas.**

◻ 4 Completamente de acuerdo ◻ 3 Parcialmente de acuerdo ◻ 2 Parcialmente en desacuerdo ◻ 1 Completamente en desacuerdo

| Comentarios |
| --- |

1. **Un dron con sistema de megafonía permite solicitar a todas las personas involucradas en la emergencia que puedan andar (denominadas by-standers) que permanezcan en pie, junto a otras que no puedan hacerlo, para intentar socorrerlas, si se consideran capacitados para ello.**

◻ 4 Completamente de acuerdo ◻ 3 Parcialmente de acuerdo ◻ 2 Parcialmente en desacuerdo ◻ 1 Completamente en desacuerdo

| Comentarios |
| --- |

1. **Un dron con sistema de megafonía permite indicar instrucciones para autoprotegerse a personas que no son capaces de andar.**

◻ 4 Completamente de acuerdo ◻ 3 Parcialmente de acuerdo ◻ 2 Parcialmente en desacuerdo ◻ 1 Completamente en desacuerdo

| Comentarios |
| --- |

1. **Un dron con sistema de megafonía permite dar instrucciones a un by-stander de colocar a un paciente en posición lateral de seguridad o a aplicar (o autoaplicarse) medidas de hemostasia en heridas potencialmente exanguinantes.**

◻ 4 Completamente de acuerdo ◻ 3 Parcialmente de acuerdo ◻ 2 Parcialmente en desacuerdo ◻ 1 Completamente en desacuerdoº

| Comentarios |
| --- |

1. **Un dron puede transportar material sanitario útil para ayudar en la situación de emergencia como medicación (autoinyectores antídoto, analgesia), kit para hemorragias, sábana isotérmica…previo a la llegada al lugar de los primeros intervinientes a el lugar.**

◻ 4 Completamente de acuerdo ◻ 3 Parcialmente de acuerdo ◻ 2 Parcialmente en desacuerdo ◻ 1 Completamente en desacuerdo

| Comentarios |
| --- |

1. **Un dron desplegado en un escenario de emergencia sanitaria compleja permite la evaluación individual de pacientes y personas heridas y la asignación de prioridad para su atención por medio de las imágenes aportadas por la cámara y la respuesta a los mensajes de megafonía.**

◻ 4 Completamente de acuerdo ◻ 3 Parcialmente de acuerdo ◻ 2 Parcialmente en desacuerdo ◻ 1 Completamente en desacuerdo

| Comentarios |
| --- |

**SECCIÓN 2**

**Valoración del algoritmo del Sistema de Triaje Aéreo a Distancia (ARTS)**

Por favor, muestre su grado de acuerdo con los siguientes enunciados del Sistema de Triaje Aéreo a distancia mediante la escala mostrada tras los mismos. Sería muy importante que pudiera razonar su desacuerdo parcial o total con los distintos enunciados, aportando sus sugerencias de mejora, en los campos para comentarios que se sitúan tras cada enunciado.

A continuación, se muestra el algoritmo completo para que le sirva de guía en sus respuestas. La valoración a través del dron finalizará al ser asignado al paciente el
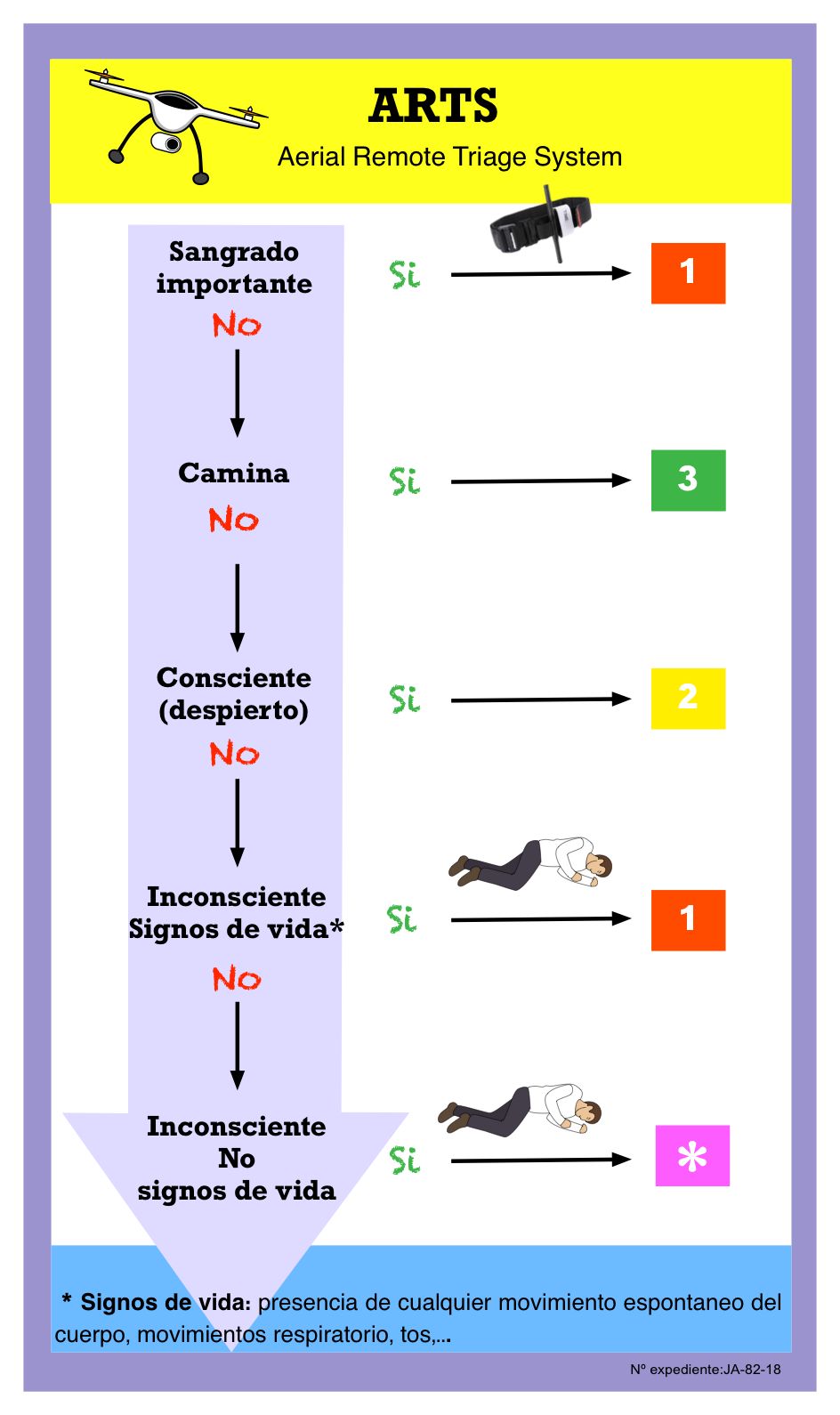
color que le corresponde según este algoritmo.

1. **El primer paso antes de iniciar el triaje será emitir un mensaje de audio a través de la megafonía del dron indicando a las personas que puedan andar que permanezcan de pie y a aquellas que no puedan deambular que realicen algún movimiento.**

◻ 4 Completamente de acuerdo ◻ 3 Parcialmente de acuerdo ◻ 2 Parcialmente en desacuerdo ◻ 1 Completamente en desacuerdo

| Comentarios |
| --- |

1. **La evaluación de las pacientes para asignar prioridad en la atención comenzará por aquellas que no responden a los mensajes emitidos por la megafonía del dron indicando que caminen o se muevan.**

◻ 4 Completamente de acuerdo ◻ 3 Parcialmente de acuerdo ◻ 2 Parcialmente en desacuerdo ◻ 1 Completamente en desacuerdo

| Comentarios |
| --- |

1. **En el proceso de evaluación a distancia y asignación de prioridad con el apoyo de drones la secuencia de aspectos a valorar sería, en este orden: 1- Sangrado importante; 2- Camina, 3- Consciente (despierto), 4- Se observa algún signo de vida.**

◻ 4 Completamente de acuerdo ◻ 3 Parcialmente de acuerdo ◻ 2 Parcialmente en desacuerdo ◻ 1 Completamente en desacuerdo

| Comentarios |
| --- |

1. **Si en la evaluación a distancia con el apoyo de drones, se observa en el paciente presencia de heridas con sangrado importante (exanguinantes según criterios de Consenso Hartford) se asignará prioridad 1 (ROJO).**

◻ 4 Completamente de acuerdo ◻ 3 Parcialmente de acuerdo ◻ 2 Parcialmente en desacuerdo ◻ 1 Completamente en desacuerdo

| Comentarios |
| --- |

1. **Si en la evaluación a distancia con el apoyo de drones, se observa en el paciente presencia de heridas con sangrado importante (exanguinantes según criterios de Consenso Hartford) se indicarán mediante los altavoces medidas de soporte básico (hemostasia), si es posible que sean aplicadas por ellos mismos o by-standers.**

◻ 4 Completamente de acuerdo ◻ 3 Parcialmente de acuerdo ◻ 2 Parcialmente en desacuerdo ◻ 1 Completamente en desacuerdo

| Comentarios |
| --- |

1. **Si en la evaluación a distancia con el apoyo de drones, se observa que el paciente mantiene capacidad de deambulación se asignará prioridad 3 (VERDE).**

◻ 4 Completamente de acuerdo ◻ 3 Parcialmente de acuerdo ◻ 2 Parcialmente en desacuerdo ◻ 1 Completamente en desacuerdo

| Comentarios |
| --- |

1. **Si en la evaluación a distancia con el apoyo de drones se observa que el paciente no camina, pero sí está consciente (despierta: responde a estímulos de audio) se asignará prioridad 2 (AMARILLO).**

◻ 4 Completamente de acuerdo ◻ 3 Parcialmente de acuerdo ◻ 2 Parcialmente en desacuerdo ◻ 1 Completamente en desacuerdo

| Comentarios |
| --- |

1. **Si en la evaluación a distancia con el apoyo de drones se observa que el paciente está aparentemente inconsciente pero presenta signos de vida (presencia de cualquier movimiento espontáneo del cuerpo, movimientos respiratorios, tos…) se asignará prioridad 1 (ROJO).**

◻ 4 Completamente de acuerdo ◻ 3 Parcialmente de acuerdo ◻ 2 Parcialmente en desacuerdo ◻ 1 Completamente en desacuerdo

| Comentarios |
| --- |

1. **Si en la evaluación a distancia con el apoyo de drones se observa que el paciente está aparentemente inconsciente y con signos de vida se indicará por altavoces que sea colocada en posición lateral de seguridad, si es posible y hay algún by-stander que pueda hacerlo.**

◻ 4 Completamente de acuerdo ◻ 3 Parcialmente de acuerdo ◻ 2 Parcialmente en desacuerdo ◻ 1 Completamente en desacuerdo

| Comentarios |
| --- |

1. **Si en la evaluación a distancia con el apoyo de drones observamos un paciente aparentemente inconsciente sin signos de vida (algún movimiento espontáneo del cuerpo, movimientos respiratorios, tos…) se asignará la prioridad * (VIOLETA).**

◻ 4 Completamente de acuerdo ◻ 3 Parcialmente de acuerdo ◻ 2 Parcialmente en desacuerdo ◻ 1 Completamente en desacuerdo

| Comentarios |
| --- |

1. **En el proceso de evaluación a distancia y asignación de prioridad con el apoyo de drones, se asigna la PRIORIDAD * (color VIOLETA) indicando que el paciente queda en una situación de espera para ser reevaluada in situ por los primeros intervinientes.**

◻ 4 Completamente de acuerdo ◻ 3 Parcialmente de acuerdo ◻ 2 Parcialmente en desacuerdo ◻ 1 Completamente en desacuerdo

| Comentarios |
| --- |

1. **Si en la evaluación a distancia con el apoyo de drones no se pueden observar signos de vida en el paciente (algún movimiento espontáneo del cuerpo, movimientos respiratorios, tos…) se indicará por altavoces que sea colocada en posición lateral de seguridad, si es posible y hay algún by-stander que pueda hacerlo.**

◻ 4 Completamente de acuerdo ◻ 3 Parcialmente de acuerdo ◻ 2 Parcialmente en desacuerdo ◻ 1 Completamente en desacuerdo

| Comentarios |
| --- |

1. **La comunicación a los primeros intervinientes de los resultados del Triaje Aéreo a Distancia con Drones, previo a su acceso al lugar, puede ser de gran utilidad y ayuda.**

◻ 4 Completamente de acuerdo ◻ 3 Parcialmente de acuerdo ◻ 2 Parcialmente en desacuerdo ◻ 1 Completamente en desacuerdo

| Comentarios |
| --- |
