# Supplementary material for: Development of the Aerial Remote Triage System using drones in mass casualty scenarios: A survey of international experts
Source: PLoS One. 2021 May 11;16(5):e0242947. doi: 10.1371/journal.pone.0242947 (PMC8112676; doi:10.1371/journal.pone.0242947)
Supplement: S2 Appendix — (DOCX) [file pone.0242947.s002.docx]

**S2 Appendix. Questionnaire for experts: images assessment**

**English version**

Dear colleague,

Following the development of our project **Drones for Triage in Medical Emergencies**, we have prepared some videos with images filmed with a dron in order to really see if our theoretical algorithm is practical in simulated situations.

Your collaboration would be of great help to us at this point due to your experience and knowledge, which was very relevant in the previous phase of the theoretical evaluation of the algorithm.

Below are seven videos, with a maximum duration of 30 seconds, showing footage taken with a drone camera in a simulated emergency situation. We would like you to rate on a scale of 1 to 5 how useful the drone would be in these cases, or how likely the interaction with victims or by-standers would be, as the case may be, considering your judgement based on the images provided. We recommend that the survey be completed on a computer, in order to have a larger screen to view the images sufficiently clearly.

If you have any questions or suggestions, please contact the following email address: cagarcia@ujaen.es

Estimated time: 5 minutes, thank you very much for your collaboration!


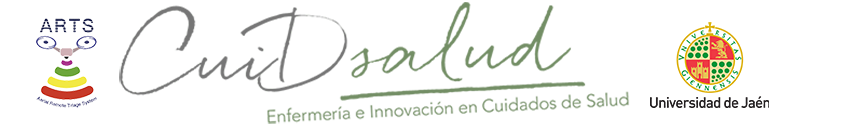


**Scene size up**

1. After watching the following video (<https://youtu.be/J__IjFWJRQQ>), how useful is a drone for the initial assessment of a scenario in an emergency situation:

□ 1. Very useless □ 2. Useless □ 3. Neutral □ 4. Useful □ 5. Very useful

**Recruitment**

1. After watching the following video (<https://youtu.be/rHFJvE5S6vA>), how likely do you think it is that bystanders could be recruited in an emergency situation using an on-board loudspeaker system on a drone:

□ 1. Highly unlikely □ 2. Unlikely □ 3. Neutral □ 4. Likely □ 5. Highly likely

**Bleeding assessment**

1. After watching the following video (<https://youtu.be/hkSq3Oq4pp4>), how useful a drone is in assessing an exsanguinating wound:

□ 1. Very useless □ 2. Useless □ 3. Neutral □ 4. Useful □ 5. Very useful

**Manual compression**

1. After watching the following video (<https://youtu.be/eKKarqGX7lY>), how likely do you think it is that the patient/bystander will follow the instructions broadcast over a drone-borne loudspeaker system to manually compress the wound:

□ 1. Highly unlikely □ 2. Unlikely □ 3. Neutral □ 4. Likely □ 5. Highly likely

**Consciousness assessment**

1. After watching the following video (<https://youtu.be/Jn6fp5K68WI>), how useful a drone is to identify signs of life such as voluntary movements in a conscious patient:

□ 1. Very useless □ 2. Useless □ 3. Neutral □ 4. Useful □ 5. Very useful

**Life sign assessment**

1. After watching the following video (<https://youtu.be/wWzHmLdWg7k>), how useful a drone is in identifying signs of life such as respiratory movements in an unconscious patient:

□ 1. Very useless □ 2. Useless □ 3. Neutral □ 4. Useful □ 5. Very useful

**Recovery position**

1. After watching the following video (<https://youtu.be/1-m-7AHN2Ls>), how likely do you think it is that the bystander will follow the instructions for positioning the patient in the recovery position:

□ 1. Highly unlikely □ 2. Unlikely □ 3. Neutral □ 4. Likely □ 5. Highly likely

| Comments |
| --- |

Thank you very much for your time!

**Spanish version**

Estimado/a compañero/a,

Siguiendo con el desarrollo de nuestro proyecto Drones para el Triaje en Emergencias Sanitarias, hemos preparado unos vídeos con imágenes filmadas con un dron para ver si, realmente, nuestro algoritmo teórico resulta práctico en escenarios de simulación.

Nos sería de gran ayuda su colaboración en este punto debido a su experiencia y conocimiento, que resultaron muy pertinentes en la fase previa de valoración teórica del algoritmo.

A continuación, se muestran siete videos de una duración máxima de 30 segundos, en los que se ven filmaciones realizadas con la cámara de un dron en una situación simulada de emergencia. Nos gustaría que valorara en una escala del 1 al 5 cómo de útil sería el dron en esos casos, o cómo de probable sería la interacción con pacientes o by-standers, según el caso, de acuerdo con su criterio tras visualizar las imágenes proporcionadas. Recomendamos que la encuesta se complete desde un ordenador, a efecto de disponer de una pantalla de mayor tamaño que permita apreciar con suficiente nitidez las imágenes.

Para cualquier duda o sugerencia puede dirigirse a la siguiente dirección de correo electrónico: cagarcia@ujaen.es

Tiempo estimado: 5 minutos. ¡Muchas gracias por su colaboración!


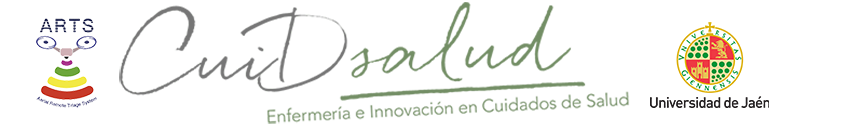


**Valoración del escenario**

1. **Tras visualizar el siguiente vídeo (**<https://youtu.be/ukxAAuvv8cg>**), cómo de útil considera un dron para la valoración inicial de un escenario en una situación de emergencia:**

□ 1. Muy inútil □ 2. Inútil □ 3. No estoy seguro □ 4. Útil □ 5. Muy útil

**Reclutamiento**

1. **Tras visualizar el siguiente vídeo (**<https://youtu.be/dBWFPO2CoKo>**), cómo de probable considera que se puedan reclutar bystanders en una situación de emergencia empleando un sistema de megafonía embarcado en un dron:**

□ 1. Muy improbable □ 2. Improbable □ 3. No estoy seguro □ 4. Probable □ 5. Muy probable

**Evaluación del sangrado**

1. **Tras visualizar el siguiente vídeo (**<https://youtu.be/rz9-t5Ez3mA>**), cómo de útil considera un dron para valorar una herida exanguinante:**

□ 1. Muy inútil □ 2. Inútil □ 3. No estoy seguro □ 4. Útil □ 5. Muy útil

**Compresión manual**

1. **Tras visualizar el siguiente vídeo (**<https://youtu.be/8yb8FynithY>**), cómo de probable considera que el paciente/bystander siga las instrucciones emitidas a través de un sistema de megafonía embarcado en un dron de comprimir manualmente la herida:**

□ 1. Muy improbable □ 2. Improbable □ 3. No estoy seguro □ 4. Probable □ 5. Muy probable

**Valoración de la consciencia**

1. **Tras visualizar el siguiente vídeo (**<https://youtu.be/-bsOWE1RhwA>**), cómo de útil considera un dron para identificar signos de vida como movimientos voluntarios en un paciente consciente:**

□ 1. Muy inútil □ 2. Inútil □ 3. No estoy seguro □ 4. Útil □ 5. Muy útil

**Valoración signos de vida**

1. **Tras visualizar el siguiente vídeo (**<https://youtu.be/6YX3nLrpcG0>**), cómo de útil considera un dron para identificar signos de vida como movimientos respiratorios en un paciente inconsciente:**

□ 1. Muy inútil □ 2. Inútil □ 3. No estoy seguro □ 4. Útil □ 5. Muy útil

**Posición lateral de seguridad**

1. **Tras visualizar el siguiente vídeo (**<https://youtu.be/FIAJZbeO2PM>**), cómo de probable considera que la bystander siga las instrucciones emitidas a través de un sistema de megafonía embarcado en un dron de colocación del paciente en posición lateral de seguridad:**

□ 1. Muy improbable □ 2. Improbable □ 3. No estoy seguro □ 4. Probable □ 5. Muy probable

| Comentarios |
| --- |

**¡Muchas gracias por su tiempo!**
