# Supplementary material for: Development of the Aerial Remote Triage System using drones in mass casualty scenarios: A survey of international experts
Source: PLoS One. 2021 May 11;16(5):e0242947. doi: 10.1371/journal.pone.0242947 (PMC8112676; doi:10.1371/journal.pone.0242947)
Supplement: Attachment — Submitted filename: Response to Reviewers.docx [file pone.0242947.s003.docx]

**Response to Reviewers**

| **Reviewer Comment** | **Author Response to Comment** | **Page number** |
| --- | --- | --- |
| 1. Please ensure that your manuscript meets PLOS ONE´s style requirements, including those for file naming. | Revised | All the manuscript |
| 2. We suggest you thoroughly copyedit your manuscript for language usage, spelling, and grammar. If you do not know anyone who can help you do this, you may wish to consider employing a professional scientific editing service. | The manuscript has been carefully revised for grammar and language and all the mistakes corrected.  The manuscript was professionally proofread. A certificate is attached. | All the manuscript |
| 3. Please provide additional details regarding participant consent.   In the ethics statement in the Methods and online submission information, please ensure that you have specified (i) whether consent was informed and (ii) what type you obtained (for instance, written or verbal, and if verbal, how it was documented and witnessed). If the need for consent was waived by the ethics committee, please include this information.” | We have added more information about the participants in the survey.  “They were first contacted by e-mail or telephone, asking for verbal consent to participate.”  “The IRB of University of Jaen made the exemption from ethical approval for this study, because no personal data were recorded.” | Page 4 |
| 4. In your Methods section, please provide additional information about the participant recruitment method and the demographic details of your participants. Please ensure you have provided sufficient details to replicate the analyses such as:   a) a description of any inclusion/exclusion criteria that were applied to participant recruitment,  b) descriptions of where participants were recruited and where the research took place. | We have expanded the information about the process of contacting participants and the inclusion criteria, in the methods section  “The ARTS algorithm was evaluated by a panel of experts through an online survey. The experts were intentionally recruited by searching for health professionals who have published on the use of drones in healthcare or in health emergencies. The panel included professionals from different disciplines, different settings (academic and healthcare), and location (Spain and other countries).” | Page 4 |
| 5. Please include additional information regarding the survey or questionnaire used in the study and ensure that you have provided sufficient details that others could replicate the analyses.   For instance, if you developed a questionnaire as part of this study and it is not under a copyright more restrictive than CC-BY, please include a copy, in both the original language and English, as Supporting Information. | Thanks for the comment.  The basic information about the questionnaires used in the survey is provided in the manuscript (tables 2, 3 and 4). In addition, the full questionnaires used are included as supplementary material (S1 and S2 Appendixes). | Page 4 |
| 6. Please note that according to our submission guidelines (http://journals.plos.org/plosone/s/submission-guidelines), outmoded terms and potentially stigmatizing labels should be changed to more current, acceptable terminology. For example: “victims” should be changed to “patients”. | Thank you for the comment.  The term “victim” is commonly used in the literature about disasters and health emergency, to refer to any person who has suffered an accident (without any stigmatizing intention). However, throughout the text we have changed this term to "injured people" to avoid any misunderstanding.  In this context, the term “patient” could be not completely appropriate. | All the documents |
| 7. To comply with PLOS ONE submission guidelines, in your Methods section, please provide additional information regarding your statistical analyses. | The statistical method used to measure the consensus among the experts, was the content validity ratio. So, information was added regarding the use of this statistical method. | Page 6 |
| 8. We note that you have indicated that data from this study are available upon request. PLOS only allows data to be available upon request if there are legal or ethical restrictions on sharing data publicly.   b) If there are no restrictions, please upload the minimal anonymized data set necessary to replicate your study findings as either Supporting Information files or to a stable, public repository and provide us with the relevant URLs, DOIs, or accession numbers. | “The full dataset is available in Figshare.” | Page 7 |
| 9. Thank you for stating the following in the Competing Interests section:   `SCA AND JMLH are founders of Emergency Medical Drone cooperative´  a. Please confirm that this does not alter your adherence to all PLOS ONE policies on sharing data and materials, by including the following statement: “This does not alter our adherence to PLOS ONE policies on sharing data and materials.” (as detailed online in our guide for authors http://journals.plos.org/plosone/s/competing-interests).  If there are restrictions on sharing of data and/or materials, please state these. Please note that we cannot proceed with consideration of your article until this information has been declared.  b. Please include your updated Competing Interests statement in your cover letter; we will change the online submission form on your behalf. | Information regarding competing interest was added in the cover letter. |  |
| 10. The data analysis used includes the essential databases, but too few search terms are used. The search terms terrorist attack, earthquake, poison attack and the like should also be used here. | The literature review was updated following the recommendations, and five new references were added.  “The search strategy used the terms earthquake, emergency medicine, drones, terrorist attacks, triage and unmanned aerial vehicle, in English and Spanish, when appropriate, retrieving articles published from inception until January 2021. ” | Page 3 |
| 11. It is also unclear how the questionnaire was created. For this purpose, the material and methods must be used to specify how the questionnaire was created. | More information regarding questionnaires creation was included in the methods section. Besides, the questionnaire was included in the supplementary materials.  “For the first round of the survey, the form included three sections. Section 1 had some questions about respondents´ education, work field and years of experience in emergencies and disasters. Section 2 had eleven statements about the use of drones in healthcare and section 3 had thirteen statements about the ARTS triage algorithm. The statements of these sections were written based on the information obtained from the literature review.”  “For the second round, the experts were asked to watch seven short videos showing footage taken with a drone camera in simulated emergencies for the different steps of the ARTS algorithm (scene size up, recruitment, bleeding assessment, manual compression, consciousness assessment, life sign assessment and recovery position)…” | Pages 4-5 |
| 12. A statistical scientific evaluation was not carried out. In particular, no experimental study was carried out. Here one could have imagined showing the experts various pictures with injury patterns (actors, filmed by the drone) in order to determine to what extent the proposed triage is sensible and possible; such an experimental part should be reworked. | A second questionnaire was sent to the experts in which they were asked to score the usefulness of the drone in simulated situations, and a new statistical analysis was carried out. | Page 5 |
